# Identification and Characterization of a Novel Lectin from the Clam *Glycymeris yessoensis* and Its Functional Characterization under Microbial Stimulation and Environmental Stress

**DOI:** 10.3390/md19090474

**Published:** 2021-08-24

**Authors:** Tatyana O. Mizgina, Irina V. Chikalovets, Valentina I. Molchanova, Rustam H. Ziganshin, Oleg V. Chernikov

**Affiliations:** 1 G.B. Elyakov Pacific Institute of Bioorganic Chemistry, Far Eastern Branch of Russian Academy of Sciences, 690022 Vladivostok, Russia; ivchik6@mail.ru (I.V.C.); molchanova_val@mail.ru (V.I.M.); 2 School of Natural Sciences, Far Eastern Federal University, 690950 Vladivostok, Russia; 3 Shemyakin-Ovchinnikov Institute of Bioorganic Chemistry, Russian Academy of Sciences, 117997 Moscow, Russia; rustam.ziganshin@gmail.com

**Keywords:** bivalve lectins, hemolymph, microorganism binding, pattern recognition receptors

## Abstract

Lectin from the bivalve *Glycymeris yessoensis* (GYL) was purified by affinity chromatography on porcine stomach mucin–Sepharose. GYL is a dimeric protein with a molecular mass of 36 kDa, as established by SDS-PAGE and MALDI-TOF analysis, consisting of 18 kDa subunits linked by a disulfide bridge. According to circular dichroism data, GYL is a β/α-protein with the predominance of β-structure. GYL preferentially agglutinates enzyme-treated rabbit erythrocytes and recognizes glycoproteins containing O-glycosidically linked glycans, such as porcine stomach mucin (PSM), fetuin, thyroglobulin, and ovalbumin. The amino acid sequences of five segments of GYL were acquired via mass spectrometry. The sequences have no homology with other known lectins. GYL is Ca^2+^-dependent and stable over a range above a pH of 8 and temperatures up to 20 °C for 30 min. GYL is a pattern recognition receptor, as it binds common pathogen-associated molecular patterns, such as peptidoglycan, LPS, β-1,3-glucan and mannan. GYL possesses a broad microbial-binding spectrum, including Gram-positive (*Bacillus subtilis*, *Staphylococcus aureus*) and Gram-negative bacteria (*Escherichia coli*, *Vibrio proteolyticus*), but not the fungus *Candida albicans*. Expression levels of GYL in the hemolymph were significantly upregulated after bacterial challenge by *V. proteolyticus* plus environmental stress (diesel fuel). Results indicate that GYL is probably a new member of the C-type lectin family, and may be involved in the immune response of *G. yessoensis* to bacterial attack.

## 1. Introduction

Lectins are proteins that possess at least one carbohydrate recognition domain (CRD), which specifically and reversibly binds to oligosaccharides (glycans) of glycoconjugates (glycoproteins, glycolipids, and proteoglycans). There is considerable interest in glycobiology relating to marine organisms and, in recent years, many lectins from marine invertebrates have been identified, including mollusks [1,2,3,4,5,6,7]. Invertebrates, unlike vertebrates, lack antibody-mediated humoral immunity in their systems [8,9]; however, invertebrates do possess innate immunity: the host defense of mollusks and other invertebrates against pathogenic infection solely depends on various pattern recognition receptors (PRRs), which bind conserved pathogen-associated molecular pattern molecules (PAMPs) expressed among the array of carbohydrate components on the surfaces of microorganisms. The innate immune system of mollusks consists of various PRRs, such as lectins, cytokines, nitric oxide synthases, and antimicrobial peptides. Among these PRRs, lectins play key roles in various immune events, including antibacterial activity, agglutination, opsonization, and phagocytosis, or the encapsulation of foreign material [10,11].

Bivalve mollusks, which are bottom filter feeders, have proven to be adequate bioindicators of sea pollution; it is no coincidence that mussels have been chosen as an object for the monitoring of coastal waters [12]. The aquatic environment contains a wide range of biological, physical, and chemical hazards. Water is a perfect medium for bacteria and parasitic microbes. Mollusks are constantly exposed not only to pathogenic attack, but also to environmental pollution products, such as heavy metals, diesel fuel, detergents, and pesticides.

Many lectins possessing various carbohydrate specificities have been purified from the hemolymph of bivalve mollusks and characterized, with emphasis on their biological properties [13,14]. In invertebrates, hemolymph lectins play an important role in protection against many pathogens, microbes, and environmental changes [15,16,17]. Additionally, many hemolymph lectins exhibit anti-cancer properties against various cancer cell lines that express globotriaosylceramides on their cell surface [18]. The vast array of hemolymph lectins with the ability to recognize and inhibit the growth of various harmful microbes and cancer cells has prompted biochemists to study them for use medicinally and as antibacterial agents [19].

Mollusks of the genus *Glycymeris* are bivalves found all over the world in sublittoral conditions, usually inhabiting sandy and gravel sediments [20]. The genus has been studied for its commercial importance: *Glycymeris glycymeris* is industrially exploited in the northeastern Atlantic Ocean (annual catches ranged from 3000 to 7000 tonnes between 1999 and 2009) [21]. There is a small-scale but expanding fishery of other species: *Glycymeris nummaria* and *Glycymeris pilosa* in the Mediterranean; *Glycymeris* *grayana* in New South Wales, Australia; *Glycymeris vovan* and *Glycymeris scripta* in the eastern Central Atlantic; *Glycymeris ovata* in the eastern Pacific; and *Glycymeris reevei* in the Philippines; etc. [22]. Modern species of *Glycymeris* have recently been identified as promising tools for ultra-high-resolution climate reconstruction, as the genus is a cosmopolitan one with evolutionary roots in the Upper Cretaceous. Some species of *Glycymeris* can live for more than 200 years, and their shells are preserved intact, thus providing an excellent archive for the reconstruction of sea surface temperature changes on seasonal and interannual time scales [23]. Despite being a diverse genus that is distributed worldwide, there is a gap of information on the properties and biological activity of glycymerids.

Thus, the objective of this research was to report on the isolation of a novel lectin (GYL) from the hemolymph of the marine clam *Glycymeris yessoensis*, as well as to describe its biochemical properties, carbohydrate-binding specificity, and functional characterization under bacterial stimulation and environmental stress.

## 2. Results and Discussion

### 2.1. Purification of the G. yessoensis Lectin

**Figure 1:**
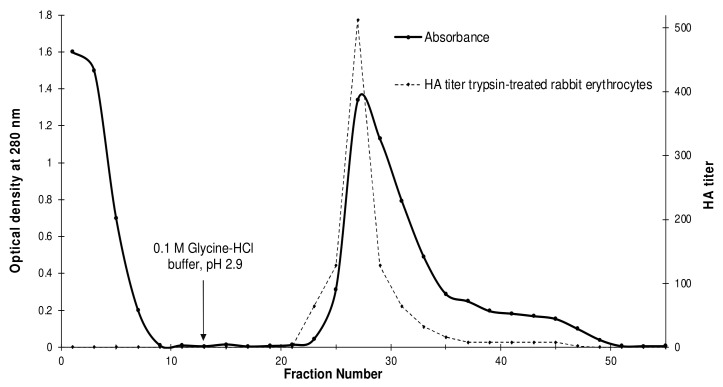
Purification of *Glycymeris yessoensis* lectin (GYL): Cell-free hemolymph (plasma) was used for affinity chromatography on a PSM–Sepharose column (2.0 × 5.0 cm) that had previously been equilibrated with 0.01 M TBS–Ca (0.01 M Tris-HCl, 0.15 M NaCl, 0.01 M CaCl_2_, pH 8.0). The elution was performed with glycine–HCl buffer, pH 2.9 (indicated by the arrow); pH was then neutralized with 1 M Tris. The elution profile (solid line) shows plasma protein (optical density at 280 nm) and agglutinating activity (titer) toward trypsin-treated rabbit erythrocytes (dotted line). A major peak and hemagglutination activity titer maximum were observed at fractions 23–35.

Affinity chromatography techniques are the most efficient isolation methods. In this study, porcine stomach mucin (PSM) was selected as the ligand for affinity sorbent synthesis because it showed the strongest inhibition of hemolymph hemagglutination using vertebrate erythrocytes in preliminary studies (data not shown). A lectin named GYL was purified from *G. yessoensis* plasma using single-step affinity chromatography on PSM–Sepharose. The elution profile of the PSM-bound fraction is shown as one peak (Figure 1). The specifically bound fraction was eluted with glycine–HCl buffer, pH 2.9.

GYL was purified 74.6-fold in a single chromatography step (Figure 1), representing 16% of the hemagglutination activity (HA) of the crude hemolymph (Table 1).

**Figure 2:**
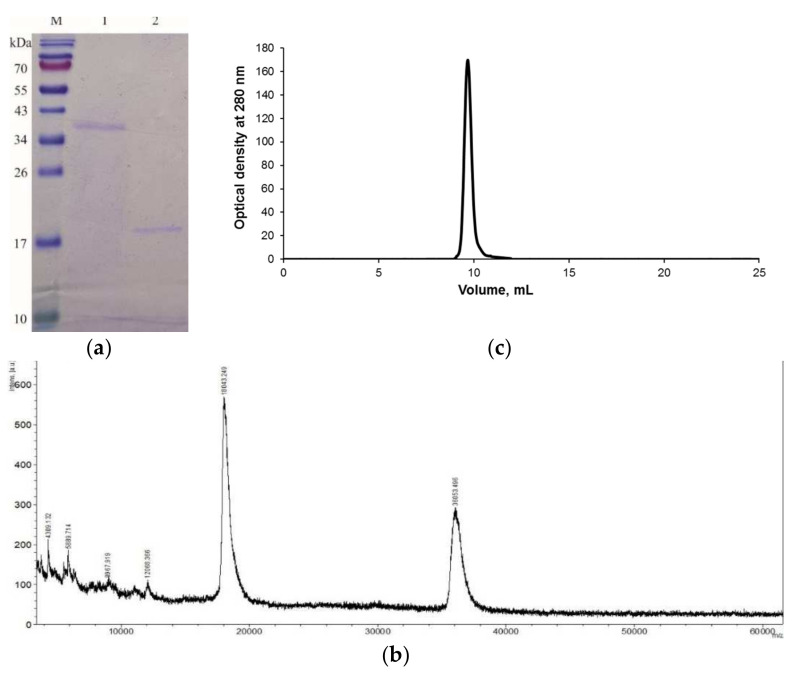
(**a**) SDS-PAGE of GYL. Protein bands were stained with Coomassie Brilliant Blue R-250 reagent. Lanes: M: molecular weight markers (kDa); 1: GYL in non-reducing conditions (without DTT); 2: GYL in reducing conditions (with DTT). (**b**) Molecular mass determination of GYL via MALDI-TOF mass spectrometry. (**c**) Size exclusion chromatography of GYL on Superdex 75.

Purified GYL demonstrated a band of 36 kDa in the absence of the reducing agent DTT, while comprising two identical subunits of 18 kDa under reducing conditions (Figure 2a). This finding indicates the presence of a disulfide bond linking two subunits with identical molecular mass.

The molecular mass of GYL was further determined by MALDI-TOF mass spectrometry. The mass spectrum contained peaks corresponding to singly charged (M+H)^+^ molecular ions from the subunit at *m*/*z* 18,118.5 Da, while the peak at *m*/*z* 36,053.5 Da corresponds to the subunit dimer (Figure 2b).

The data presented in this study are not consistent with the paradigm of hemolymph lectins as high-molecular-weight oligomers with subunits of equal or different size, held together by covalent and/or non-covalent interactions that exist in a series of distinct aggregation states. O-acetyl sialic acid-specific lectin from the hemolymph of the marine crab *Atergatis integerrimus* was shown to have a molecular mass of 216 kDa, with three subunits of 70, 72, and 74 kDa, according SDS-PAGE [24]. Mannan-binding lectin purified from cell-free hemolymph from the scallop *Chlamys farreri* has been shown to have a molecular mass of 645 kDa with a 73 kDa subunit [25]. An N-acetyl sugar-binding lectin (termed iNoL) from the slipper lobster *Ibacus novemdentatus* was composed of five subunits (330, 260, 200, 140, and 30 kDa) which, in turn, consisted of 70, 40, and 30 kDa polypeptides held together by disulfide bonds [26]. Regarding their physiological functions, bivalve lectins and other invertebrate humoral lectins play a critical role in the host defense mechanisms of mollusks, both by recognizing and binding to pathogenic microorganisms and by opsonizing for phagocytic hemocytes [11,27]. It is believed that the presence of several subunits with different types of activities provides a wide range of recognition of many foreign particles entering the hemolymph of the mollusk.

Unlike most hemolymph lectins, GYL does not form oligomers according to gel filtration on Superdex 75 (Figure 2c) and native electrophoresis (data not shown). It is possible that different biological functions are performed by several lectins circulating in the *G. yessoensis* hemolymph. Recently, we isolated mannan-binding lectin (GYLman) from the cell-free plasma of the clam *G. yessoensis* [28]. Due to the diverse carbohydrate specificity of these lectins, the recognition of a vast array of microorganisms becomes possible. Therefore, it is possible that, in the circulation of marine mollusks, multiple lectins with diverse carbohydrate specificities may also play an important role in defense by recognizing a vast array of invading microorganisms.

### 2.2. Hemagglutinating Activity and the Carbohydrate Specificity

The isolated lectin (GYL) agglutinated all erythrocytes tested, suggesting that this activity is not highly specific. Treatment of erythrocytes with trypsin was shown to enhance the hemagglutination. GYL strongly agglutinated rabbit-enzyme-treated erythrocytes, demonstrating a maximal HA titer of 512, and moderately agglutinated human and murine erythrocytes (Table 2).

GYL has unusual carbohydrate specificities that were examined via hemagglutination inhibition test. The GYL HA was not inhibited by any of the mono-, di- or polysaccharides examined, even at the maximum concentration of 100 mM, with the exception of L-fucose—a common component of many N- and O-linked glycans and glycolipids produced by mammalian cells (Table 3).

As demonstrated by Table 3, GYL preferentially recognizes glycoproteins containing O-glycosidically linked glycans, such as PSM [29] and fetuin [30], and N-glycosidically linked glycans, including thyroglobulin [31] and ovalbumin [32]. All of these glycoproteins contain sialic acids: N-acetyl neuraminic acid or N-glycolylneuraminic acid. Since sialidase treatment of sialoglycoproteins resulted in a substantial increase in the affinity of the lectin to desialylated glycoproteins, it is likely that GYL recognizes O- and N-linked carbohydrate chains, and interacts with terminal Gal residues. N-glycosylated proteins, such as α1-acid glycoprotein [33] and ovomucoid [34], showed no capacity to interact with GYL. It is interesting to note that most lectins from the hemolymph of invertebrates have a preference for sialic acid—especially among the arthropods [24,35,36]. Humoral lectins from several marine bivalves exhibit a particular pattern of specificity towards multivalent glycoproteins, such as PSM and asialo-PSM, interacting with either the terminal Gal/GalNAc or the core GalNAc that is attached to the Ser/Tre residue in the protein part of the glycoconjugate [37,38].

### 2.3. Physical and Chemical Characterization of GYL

**Figure 3:**
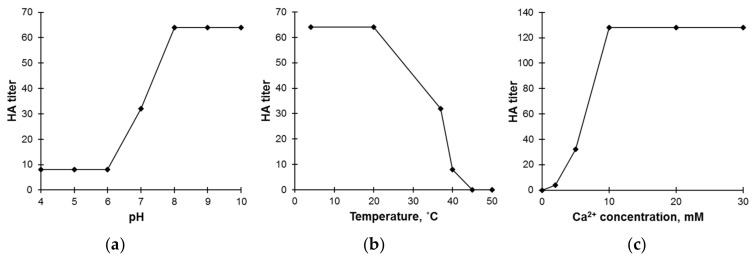
Effects of (**a**) pH, (**b**) temperature, and (**c**) CaCl_2_ on the hemagglutination activity of GYL.

After exposing GYL to various pH values for 1 h, we noted the optimal range of pH to be in the alkaline zone (Figure 3a). Increases in HA accompanied by increases in pH are also characteristic of the lectins of many invertebrates—for example, lectin from the marine sponge *Aplysina fulva* [39], the clam *R. philippinarum* [38], and the mussel *Crenomytilus grayanus* [40]. Regarding temperature, GYL retained its HA up to 20 °C; however, HA dramatically decreased at temperatures above this. The optimal activity of GYL was observed between 5 and 20 °C, corresponding to normal seawater temperatures (10–20 °C) (Figure 3b).

Loss of HA when samples are heated above 50–60 °C is characteristic of lectins of marine bivalve mollusks. The HA of lectin from the mussel *C. grayanus* was fully maintained after heating at 50 °C [40]. Meanwhile, in the case of bivalve lectins from *Meretrix lusoria* and *Corbicula fluminea*, the highest HA values were observed at 4–10 °C [41]. The Manila clam lectin (MCL) from *R. philippinarum* was fully active at normal seawater temperatures of 10–20 °C [38]. GYL may belong to the family of C-type lectins, since its HA was abolished by demetallization. This effect was reversible, as the addition of CaCl_2_ to metal-free GYL fully restored its activity (Figure 3c). However, since it is still unclear whether GYL protein contains the C-type CRD motif, it is somewhat premature to describe this lectin as a C-type lectin.

### 2.4. Structural Studies by Circular Dichroism

The content of secondary structure that is present in any protein can be determined by analyzing its far-UV circular dichroism (CD) spectrum as a sum of the fractional multiples of such reference spectra for each structural type. The CD spectrum of native GYL (pH 8.5, 25 °C) in the far-UV region (190–260 nm) showed a broad negative band centered around 215 nm and a positive maximum near 192 nm. Using the method of Sreerama, estimates of the secondary structure of lectin showed that a characteristic feature of GYL was the predominance of β-structure (Table 4). This secondary structure is quite similar to the previously reported structures of other lectins from marine invertebrates, including lectins from other bivalves. For example, the secondary structure of *Crenomytilus grayanus* lectin [42] consists of 43.1% β-structures and 4.3% α-helices, while MCL [43] consists of 60.7% β-structures and 6.2% α-helices, and the secondary structure of *Mytilus trossulus* lectin consists of 61.4% β-structures and 6% α-helices [44].

### 2.5. Amino Acid Sequencing of GYL

Attempts to establish the N-terminal sequence of GYL were unsuccessful. The lectin was, therefore, presumed to have a blocked amino-terminus, which is characteristic of marine invertebrate lectins [45], and the amino acid sequence of GYL was determined by tandem mass spectrometry. GYL was digested with trypsin and analyzed with nano-ESI MS/MS. Five peptides were sequenced de novo and applied for BlastX analysis (http://www.ncbi.nlm.nih.gov/BLAST/, accessed on 26 February 2021); these peptides showed no significant homology with other lectins listed in the BLAST (Table 5). The National Center for Biotechnology Information (NCBI) Conserved Domain Search program identified conserved EPN (Glu-Pro-Asn) and WND (Trp- Asn- Asp) motifs in the GYL peptide sequence, which are characteristic of the CRD of C-type lectins (CTL). There are four Ca^2+^-binding sites in this CRD. In vertebrates, the first motif is always EPN or QPD (Gln-Pro-Asp), while the second motif is always WND. These motifs seem to be more various in molluscan CTL; currently, at least 7 variations in the first motif and more than 10 variations of the second motif have been presented in different Mollusca [11]. Our results indicate that GYL is probably a new member of the CTL family, and may be involved in the immune response of *G. yessoensis* to bacterial attack.

### 2.6. Tissue Distribution of GYL

**Figure 4:**
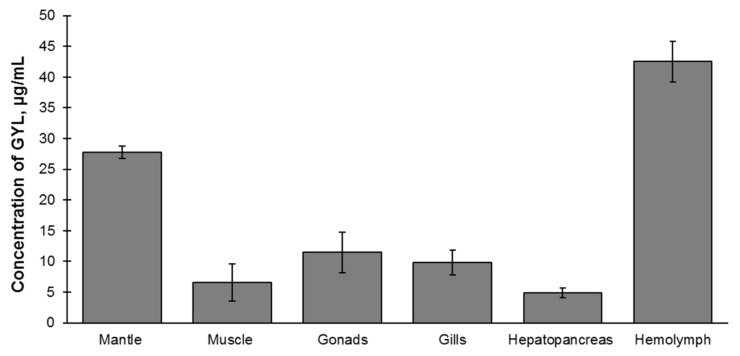
Distribution of GYL in different tissues and hemolymph of *G. yessoensis* measured by ELISA. Four biological replicates were performed, and the data are shown as mean ± S.D (*n* = 3).

It is extremely important to determine the distribution of lectins in tissues in order to understand their function. Quantitative enzyme-linked immunosorbent assay (ELISA) made it possible to establish that GYL is widely expressed throughout the tissues of healthy clams; however, levels of the lectin in the hemolymph and mantle were found to be 3.5- and 2.4-fold greater, respectively, than that found in the gonads. By contrast, expression levels of GYL were much lower in the other tested tissues, including gill, muscle, and hepatopancreas (Figure 4).

Considering that bivalve mollusks completely expose themselves to the aquatic environment, which contains a large number of pathogenic microorganisms, bivalve lectins have mainly been identified in the hemolymph and hemocytes; however, these lectins are also present in different parts of the body, including the gill, gonad, mantle and foot muscle tissues [8]. Similar data were observed in our study, which revealed that GYL was highly expressed in the hemolymph and mantle [13,46]. The hemolymph of bivalve mollusks is a protective and transport circulatory system in the composition of tissues of the internal environment, which is largely responsible for maintaining homeostasis and the formation of physiological adaptations of animals to changing habitat conditions. For marine bivalves, the mantle is the tissue involved in a continuous water exchange, and is therefore more susceptible to pathogenic infection [47].

Humoral lectins have been proposed to act as hemolysins, opsonins, and sugar-specific antibody-like molecules in invertebrates. The clam *Ruditapes philippinarum* has been observed to produce a lectin—named MCL—in hemocytes when infected with the protozoan parasite *Perkins olseni*. Recently synthesized MCL is then secreted into the hemolymph as a precursor protein with a molecular mass of 74 kDa. Researchers noticed that, with a higher degree of infection, higher hemagglutination activity occurred, resulting in hemagglutination titers greater than 1000-fold that of the uninfected mollusk [48]. Humoral lectin (CqL) isolated from the crayfish *Cherax quadricarinatus* has been shown to participate in the immune mechanisms of crayfish challenged with LPS or β-glucan. The specific activity of CqL challenged with β-glucan was 250% and 160% higher than that of the control group following 2 and 6 h of stimulation, respectively [49].

### 2.7. Binding of GYL to PAMPs and Microorganisms

**Figure 5:**
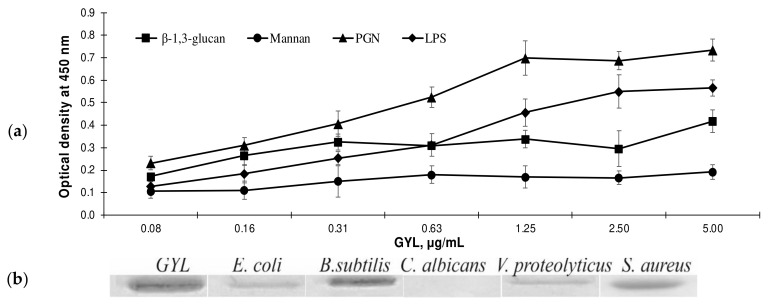
(**a**) ELLA analysis of the interaction between GYL and various PAMPs. The data are the mean ± SD (*n* = 3). (**b**) The microbe binding activity of GYL as revealed by Western blot.

The wide distribution of GYL in the hemolymph and various other parts of the body suggests that this lectin plays an important role in the immune defense of the clam against pathogenic microorganism infections. Many C-type lectins bind to components of the microbial cell wall—mainly various carbohydrate groups of these molecules—and trigger an innate immune response. Enzyme-linked lectin assay (ELLA) has been used to test the interactions between lectins and the microorganism components known as PAMPs. GYL preferentially bound peptidoglycan (PGN) and LPS, but had little binding activity toward β-1,3-glucan and mannan, which are usual components of the yeast cell wall. Additionally, these values increased with corresponding increases of GYL concentration, suggesting that the binding activities of GYL towards these examined PAMPs exhibit dose-dependent effects (Figure 5a). A sialic-acid-binding lectin (SABL) from the manila clam *Ruditapes philippinarum* displayed apparent binding activity towards LPS and PGN, but not to glucan, and exhibited obvious agglutination activities against the Gram-positive bacterium *Vibrio harveyi* [50]. The CTL from the oyster *Crassostrea gigas* (designated as CgCLec-2) could bind various PAMPs, including LPS, mannan, and PGN. CgCLec-2 exhibited growth suppression activity against *Staphylococcus aureus* and enhanced the phagocytic activity hemocytes to *Vibrio splendidus* [51].

Direct interactions of lectin with bacterial cells were assessed via Western blot using conjugate IgG_GYL_–HRP (horseradish peroxidase). After incubation of microbes with lectin, we examined lectin content in the homogenate following destruction and sedimentation of the cell pellet. GYL possessed a broad microbial-binding spectrum, binding all tested types of microbe, including Gram-positive bacteria (*B. subtilis*, *S. aureus*) and Gram-negative bacteria (*E. coli*, *V. proteolyticus*), but not the fungus *C. albicans* (Figure 5b).

From our results, GYL demonstrated stronger interactions with *S. aureus* and *B. subtilis* compared to other microbes, which corresponds to the binding spectra of GYL with PAMPs. PGN, present in Gram-negative bacteria, is known to comprise only 10–20% of their cell wall components, compared with 50–80% in Gram-positive bacteria. Additionally, the PGN of gram-negative bacteria is buried in the periplasmic space, while the PGN of gram-positive bacteria is exposed at the surface. The ability of C-type lectins to recognize microorganisms is largely dependent on the density of PAMPs present on the microbial surface [52]. Our data suggest that GYL recognizes microorganisms and interacts directly with the cell wall components of these microorganisms, such as PGN from Gram-positive bacteria and LPS from Gram-negative bacteria, indicating that GYL may serve as a PRR.

### 2.8. Temporal Levels of GYL after Microbial Challenge and Anthropogenic Pollution

Various immunological functions of C-type lectins have been shown in mollusks in recent decades. The expression level of most lectins is increased after stimulation by microorganisms or PAMPs, indicating that their participation in the immune response to various pathogens works via specific PAMP recognition [11,53].

**Figure 6:**
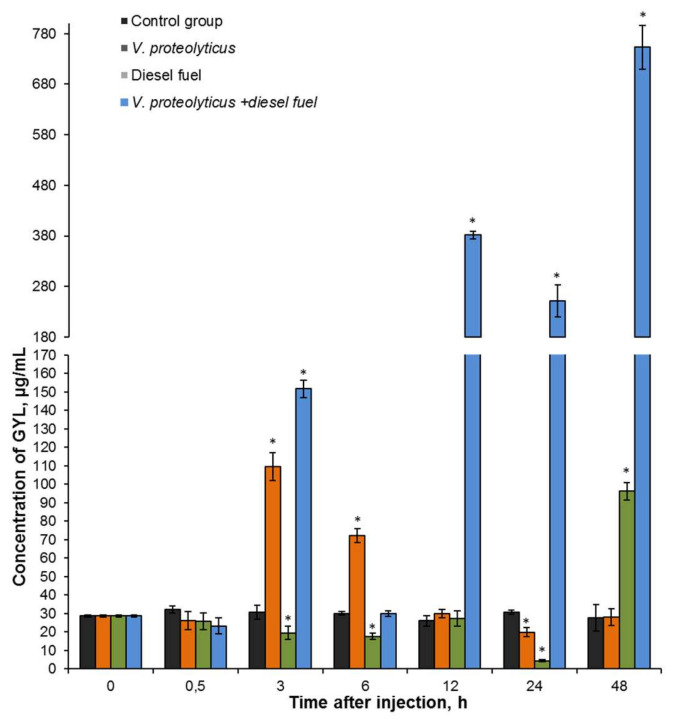
Temporal expression levels of GYL in the hemolymph after *V. proteolyticus* challenge and diesel fuel exposure as measured by ELISA. Vertical bars represent the mean ± SD (*n* = 3). Significant (*p* < 0.05) differences from the control are indicated with asterisk.

Temporal expression levels of GYL in the hemolymph following *V. proteolyticus* stimulation and diesel fuel exposure were investigated using ELISA. Compared to the control group, expression levels of the lectin were increased in the *V. proteolyticus* challenge group, reaching a maximum at 3 h (about 3.6-fold) post-injection, thus suggesting activation of defenses against the invading pathogen. The decreases seen in GYL levels 12–24 h post-challenge may be due to the inhibition of GYL synthesis in response to the bacterial infection. However, the increasing expression to 48 h evidences a recovery of the immune response (Figure 6). Similar results have been reported for the galectin from the red abalone *Haliotis rufescens*, where a peak in expression was observed 3 h post-challenge with *V. alginolyticus* [54], as well as in the clam *Tegillarca granosa* exposed to *V. parahaemolyticus* [55] and the bivalve *M. trossulus* post-challenge using *V. proteolyticus* [45]. No significant variation in expression levels was observed in the group exposed to diesel fuel alone (Figure 6). Unexpected results were obtained for the clams after microbial challenge plus exposure to diesel fuel. The expression levels of lectin increased about 25-fold at 48 h post-injection, suggesting that bacterial infection and anthropogenic factors had a significant effect on the mollusks, causing them to increase the synthesis of protective molecules.

Investigation of the characteristics of the immune system of the mussel *Mytilus edulis* in response to oil pollution has shown a cyclic pattern of the resulting immune suppression, characterized by an adaptation dose–response curve [56]. During exposure of the mussel *M. trossulus* to a synthetic detergent or diesel fuel, changes in lectin content were dependent on the time of exposure [57]. Over 36 h of exposure, diesel fuel, at a concentration of 2 mL/L, induced biochemical and cytological antistress mechanisms in the bodies of three species of Black Sea bivalves (*Anadara inaequivalvis*, *Mytilus galloprovincialis*, and *Chamelea gallina*) [58].

The growing variety of pollutants released into the aquatic environment requires the development of reliable and affordable strategies for toxicity testing and biota monitoring in aquatic ecotoxicology. Environmental stress can cause additional sublethal effects, such as mutations and epigenetic signatures, affecting offspring through germline-mediated transgender inheritance, the formation of phenotypic plasticity, increased susceptibility to disease, tissue pathology, changes in social behavior, and biological invasions [59].

Various quantitative and qualitative characteristics of hemocyte populations are most often used as indicators of environmental or experimentally induced stress in mollusks; these include the number of circulating cells, the relationship between different types of cells, cell morphology, phagocytic and other cellular reactions, and several biochemical indicators [60]. 

Determining the levels of change of lectins in response to anthropogenic contaminants may be a useful marker for the state of the immune system of bivalves, especially alongside other characteristics of the defense systems of marine invertebrates. Further research is needed in order to develop a multi-assay approach for monitoring environmental pollution using lectins.

## 3. Materials and Methods

### 3.1. Materials

Monosaccharides were obtained from Merck (Darmstadt, Germany). Porcine stomach mucin type III (PSM), bovine serum albumin (BSA), thyroglobulin, and trypsin were purchased from Sigma-Aldrich (St. Louis, MO, USA). 3,3′-Diaminobenzidine (DAB) was obtained from AppliChem (Darmstadt, Germany). LPS from *E. coli* Serotype 055:B5, peptidoglycan (PGN) from *S. aureus*, β-1,3-glucan from *Euglena gracilis*, and mannan from *S. cerevisiae* were purchased from Sigma-Aldrich (St. Louis, MO, USA). CNBr-activated Sepharose 4B was purchased from GE Life Sciences (Uppsala, Sweden). Centricon Ultracel YM-10 was purchased from Millipore (Carrigtwohill, Ireland). Human erythrocytes were obtained as outdated red cell concentrates from the Centre of Blood Utilization (Vladivostok, Russia). Rabbit and mouse erythrocytes were obtained from the vivarium of the G.B. Elyakov Pacific Institute of Bioorganic Chemistry (Vladivostok, Russia). The standard proteins used for apparent molecular mass estimation by SDS-PAGE were purchased from Thermo FS (Vilnius, Lithuania). Coomassie Brilliant Blue R-250 was purchased from Sigma-Aldrich (St. Louis, MO, USA). Polyvinylidene difluoride (PVDF) membrane and Trans-Blot Turbo system were purchased from Bio-Rad (Singapore). Gram-positive (*S. aureus* KMM 434, *B. subtilis* ATCC 6633) and Gram-negative (*E. coli* VKPM V 7335, and *V. proteolyticus* CCUG 20302T) bacteria and the yeast *C. albicans* KMM 455 were obtained from the Collection of Marine Microorganisms (KMM) of the G.B. Elyakov Pacific Institute of Bioorganic Chemistry, Far Eastern Branch of the Russian Academy of Sciences (Vladivostok, Russia).

### 3.2. Collection of Hemolymph

For experiments, *G. yessoensis* clams ~50 mm in shell length and 23–25 g in weight were collected at the Marine Experimental Station of the G.B. Elyakov Pacific Institute of Bioorganic Chemistry in Troitsy Bay (Posyet Bay, Sea of Japan). The hemolymph from the *G. yessoensis* clams was assembled by dissecting the adductor muscle and was centrifuged to separate hemocytes at 1500 rpm for 1 h at 4 °C, and at 30,000 rpm for 1 h at 4 °C to lighten the hemolymph. The clear supernatant (plasma) was tested for agglutinated activity and was stored frozen at −20 °C for further use.

### 3.3. Isolation and Purification of Lectin from G. yessoensis (GYL)

For the isolation of GYL by affinity chromatography, 100 mg PSM was coupled with CNBr-activated Sepharose 4B according to the manufacturer’s instructions. The clear supernatant (50 mL) was applied to a PSM-Sepharose column (2.0 × 5.0 cm), which was previously equilibrated and eluted with 0.01 M TBS-Ca (0.01 M Tris-HCl, 0.15 M NaCl, 0.01 M CaCl_2_, pH 8.0). After the elution of unbound proteins from the column with 0.01M TBS–Ca until the absorbance of the effluent at 280 nm was zero, the specifically bound fraction was then eluted with glycine-HCl buffer (0.1 M glycine, pH 2.9); the pH was then neutralized with 1 M Tris. The active fractions were pooled, concentrated via ultrafiltration, dialyzed against distilled water for 36 h, and freeze-dried to yield GYL.

### 3.4. Molecular Mass Determination

The relative molecular mass of the purified GYL subunit was estimated by SDS-PAGE using 15% separation and 4% stacking gels [61] in the absence and presence of 10 mM dithiothreitol (DTT). The molecular weight was further investigated by mass spectrometry on an Ultraflex III MALDI-TOF/TOF.

### 3.5. Size Exclusion Chromatography of GYL

Approximately 250 μg of GYL was subjected to size exclusion chromatography with a Superdex 75 Increase 10/30 GL (GE Healthcare, Uppsala, Sweden) equilibrated in 0.01 M TBS–Ca, pH 8.5, using an AKTA FPLC chromatograph (Amersham Pharmacia Biotech, Uppsala, Sweden). Protein was eluted with the same buffer at a flow rate of 0.5 mL/min, and the absorbance was monitored at 280 nm.

### 3.6. Preparation of 2% Suspension of Native or Enzyme-Treated Erythrocytes

Erythrocytes were washed five times with 50 volumes of 0.15 M NaCl. After washing, a 2% erythrocyte suspension (*v*/*v*) was prepared in 0.15 M NaCl and used as native erythrocytes. Trypsin-treated erythrocytes were prepared as follows: one-tenth volume of 0.5 % (*w*/*v*) trypsin solution was added to a 2% native erythrocyte suspension, and the mixture was incubated at 37 °C for 2 h. After incubation, the erythrocytes were washed five times with saline, and a 2% suspension (*v*/*v*) of trypsin-treated erythrocytes was prepared in saline.

### 3.7. Hemagglutinating Activity (HA) and Inhibition Assay

Hemagglutination assays and hemagglutination inhibition assays were performed as described previously [40]. Briefly, to assay the HA, a sample of GYL was two-fold serially diluted with 0.01 M TBS–Ca in the microtiter U-plates. To the sample (25 μL) in each well, an equal volume of 2% suspension of native or trypsin-treated erythrocytes was added, and the mixture was agitated. The HA was visually evaluated after 30 min. Titer values were defined as the reciprocal of the highest dilution of lectin that gave visible hemagglutination. 

For the hemagglutination inhibition assay, the aqueous solutions of various substances (glycoprotein: 1 mg/mL or sugar: 100 mM) were two-fold serially diluted with 0.01 M TBS–Ca. To each sample (25 μL), GYL (25 μL, 4 doses of agglutination) and 2% erythrocyte suspension (25 μL) were added successively, and the mixture obtained was stirred and kept for 1 h. The minimal concentration of each substance required for complete inhibition was determined.

### 3.8. Effect of Temperature, pH and Divalent Cations on GYL Activity

The effects of pH, temperature, and divalent cations on GYL were evaluated, as described previously [62]. Briefly, to study the effect of temperature, aliquots of the lectin (0.05 mL, 1 mg/mL) were incubated at 4, 20, 37, 40, 45, 50, and 60 °C for 30 min, and the hemagglutination assay was performed after cooling. The GYL’s pH dependence was determined by pre-incubating the aliquots of the lectin samples (0.05 mL, 1 mg/mL) with different pH buffers at 25 °C overnight: 0.01 M acetate buffer (pH 4.0–6.0), 0.01 M Tris-HCl buffer (pH 7.0; 8.0), and 0.01 M carbonate buffer (pH 9.0; 10.0). The samples were subsequently dialyzed against 0.01 M TBS–Ca to eliminate the pH effect for 12 h, after which the HA was tested. To evaluate the effect of metal ions, an aliquot of GYL (0.3 mL, 1 mg/mL) was dialyzed against a 20-fold volume of TBS with 0.02 M EDTA (ethylenediaminetetraacetic acid) 12 h and against 0.01 M TBS overnight, after which the HA was determined. CaCl_2_ was added to EDTA-treated GYL solution to a final concentration of 30 mM, and the hemagglutination titer was evaluated again.

### 3.9. Protein Content and N-Terminal Sequence

The protein content was determined according to Lowry’s method [63], using BSA as a standard. The N-terminal sequence of GYL was analyzed using Edman degradation on a Procise 492 cLC protein sequencer (Applied Biosystems, Foster City, CA, USA).

### 3.10. Circular Dichroism

The secondary structure of GYL was measured using a Jasco J-500A spectropolarimeter (Jasco, Tokyo, Japan). All spectra were analyzed in the UV ranges 190–240 nm and 240–320 nm in 1 mm and 1 cm path length cells, respectively. The protein concentration was ~0.5 mg/mL. The mean residue ellipticity was measured as a function of wavelength. The secondary structure content of the spectra was determined by the method of Sreerama using the CDPro software package program [64].

### 3.11. Amino Acid Sequencing of Lectin

#### 3.11.1. Liquid Chromatography and Mass Spectrometry

GYL sequencing was performed by digestion with trypsin and analysis of the different peptides. GYL samples were loaded onto a homemade 100 µm × 20 mm trap column, packed with Inertsil ODS3 3 µm sorbent (GLSciences) in the loading mobile phase (2% acetonitrile (ACN), 98% H_2_O, 0.1% trifluoroacetic acid) at 10 µL/min flow and separated at room temperature in a home-packed [65] 100 µm × 300 mm fused-silica column packed with ReproSil-Pur C18AQ 1.9 (Dr. Maisch) into an emitter prepared with a P2000 Laser Puller (Sutter, Novato, CA, USA). Reverse-phase chromatography was performed using an Ultimate 3000 Nano LC System (Thermo Fisher Scientific, Waltham, MA, USA), which was coupled to the Orbitrap Q Exactive Plus mass spectrometer (Thermo Fisher Scientific, Waltham, MA, USA) via a nanoelectrospray source (Thermo Fisher Scientific, Waltham, MA, USA). Water containing 0.1% (*v*/*v*) FA was used as mobile phase A, and ACN containing 0.1% FA (*v*/*v*) and 20% (*v*/*v*) H_2_O as mobile phase B. GYL peptides were eluted from the trap column with a linear gradient: 3–6% B for 3 min; 6–25% B for 30 min, 25–40% B for 25 min, 40–60% B for 4 min, 60% B for 3 min, 60–99% B for 0.1 min, 99% B for 10 min, and 99–2% B for 0.1 min, at a flow rate of 500 nL/min. After each gradient, the column was re-equilibrated with A for 10 min. MS data were collected in data-dependent acquisition (DDA) mode. MS1 parameters were as follows: 70K resolution, 350–2000 scan range, max injection time 30 s, AGC target 3 × 10^6^. Ions were isolated with a 1.4 *m*/*z* window, preferred peptide match, and isotope exclusion. Dynamic exclusion was set to 30 s. MS2 fragmentation was carried out in high-energy collisional dissociation (HCD) mode at 17.5K resolution with normalized collision energy (NCE) 29, max injection time 50 s, and AGC target 2 × 10^5^, loop count –10. Other settings: charge exclusion—unassigned, 1, > 7.

#### 3.11.2. Data Analysis

MS raw files were analyzed using PEAKS Studio 10.0 (Bioinformatics Solutions Inc., Waterloo, ON, Canada) [66]. Identification of proteins was carried out by searching against the *Mollusca* UniProt FASTA database version 26.02.2021 (438,844 entries) with a carbamidomethyl Cys as a fixed modification and deamidation Asn/Gln and Met oxidation as variable modifications. The false discovery rate for peptide spectrum matches was set to 0.01, as determined by searching a reverse database. Enzyme specificity was set as C-terminal to arginine and lysine, and a maximum of two missed cleavages were allowed in the database search. Peptide identification was performed with an allowed initial precursor mass deviation up to 10 p.p.m. and an allowed fragment mass deviation of 0.02 Da.

The obtained peptide sequences were analyzed using BlastX (http://www.ncbi.nlm.nih.gov/BLAST/, accessed on 26 February 2021).

### 3.12. Tissue Distribution of GYL

For the tissue distribution analysis of GYL, hepatopancreas, muscle, gill, gonad, hemocyte, and mantle tissues from four healthy adult clams were collected as parallel samples. The hemolymph was collected as described above. Extracts of tissues and hemolymph were obtained to analyze GYL expression levels via enzyme-linked immunosorbent assay (ELISA). Wells of microtiter plates (Nunc, Denmark) were coated with 100 µL of IgG_GYL_ (20 µg/mL) in 0.1 M carbonate buffer pH 9.5 overnight at 4 °C. Plates were emptied and washed five times with 0.01 M TBS, pH 8.0, containing 0.05% Tween-20. Then, wells were blocked with 250 µL of 1% BSA for 2 h at room temperature. After washing, a series of two-fold dilutions (100 µL) of GYL solution (from a concentration of 500 µg/mL) in 0.01 M TBS, pH 8.0, were prepared on the plate as a standard antigen, and extracts of tissues and hemolymph (100 µL) were added to other wells and incubated for 2 h at room temperature with shaking. After washing, plates were probed for 2 h at room temperature with 100 µL of IgG_GYL_ conjugated with horseradish peroxidase (IgG_GYL_-HRP), diluted 1:500. After washing, bound IgG_GYL_–HRP was detected using TMB (3,3′,5,5′-tetramethylbenzidine) as a substrate. The reaction was stopped by adding 50 µL of 2 M H_2_SO_4_ to the wells. The end product was measured at 450 nm. For each sample, experimental reactions were run in triplicate. Each sample of extract and hemolymph was normalized by protein before the experiment. 

IgG_GYL_ was obtained via the immunization of rabbits by homogeneous GYL as described by Du et al. [67]. Antiserum titer was determined by double immunodiffusion [68] using 1.5% agar in 0.01 M TBS, pH 8.0, containing 0.01% sodium azide.

IgG_NRS_ was obtained via the immunization of rabbits with normal rabbit serum (NRS).

The specificity of IgG_GYL_ was confirmed via ELISA using BSA and IgG_NRS_ as negative controls.

IgG_GYL_–HRP was prepared using the periodate method [69]; it was shown that the specificity of the conjugate is not inferior to that of the original immunoglobulins.

### 3.13. PAMP-Binding Assay

The dose-dependent PAMP-binding activity of GYL was measured via enzyme-linked lectin assay (ELLA) as described previously, with some modifications [70]. Wells of microtiter plates (Nunc, Denmark) were coated with 100 µL of PAMPs—including LPS, PGN, β-1,3-glucan, and mannan (50 µg/mL)—in 0.01 M TBS–Ca overnight at 4 °C; the same concentration of BSA was used as a control. Plates were emptied and washed five times with 0.01 M TBS–Ca containing 0.05% Tween-20. Then, wells were blocked with 250 µL of 1% BSA for 2 h at room temperature. After washing, plates were probed for 2 h at room temperature with 100 µL of GYL conjugated with horseradish peroxidase (GYL–HRP) prepared with gradient dilution from 5.0 to 0.078 µg/mL using 0.01 M TBS–Ca. After washing, bound GYL–HRP was detected using TMB as a substrate. The reaction was stopped by adding 50 µL of 2 M H_2_SO_4_ to the wells. The end product was measured at 450 nm. For each sample, experimental reactions were run in triplicate.

GYL–HRP was prepared using the periodate method [69].

### 3.14. Microbial-Binding Assay

An assay was performed as described previously, with some modifications [71]. Gram-positive (*S. aureus*, *B. subtilis*) and Gram-negative (*E. coli*, *V. proteolyticus*) bacteria and the yeast *C. albicans* were used. Cell suspensions were adjusted to OD_600_ = 0.8–1.0, which corresponds to the number of cells from 2 × 10^8^ to 2 × 10^10^ in 1 mL. To 50 μL of cell suspensions, 50 μL of lectin (200 μg/mL) in 0.01 M TBS–Ca was added by gentle orbital rotation for 1 h at room temperature. Microbes were pelleted and washed five times with 1 mL of binding buffer. Bound lectin was subsequently eluted by with 7% SDS for 1 min and, after centrifugation at 10,000 rpm, the binding spectrum of GYL was determined by Western blot, as follows: Proteins were fractionated by SDS-PAGE through 15% gel and transferred onto PVDF membrane via the Trans-Blot Turbo system. The membrane was blocked with 0.01 M TBS–Ca containing 1% BSA at 37 °C for 1 h, and incubated with IgG_GYL_–HRP diluted 1:500 at 37 °C for 1 h. The membrane was washed three times with 0.01 M TBS–Ca containing 0.05% Tween-20, and protein bands were stained with freshly prepared substrate solution containing 3,3′-diaminobenzidine (DAB) and stopped by washing with distilled water. GYL and BSA were employed as positive and negative controls, respectively

### 3.15. Temporal Levels of GYL after Microbial Challenge and Anthropogenic Pollution

For the microbial challenge experiment, *G. yessoensis* clams ~50 mm in shell length and 23–25 g in weight were collected as previously described. A total of 120 mollusks were employed, randomly divided into 4 groups (30 individuals each group), and maintained in aerated tanks with 125 liters of seawater with a salinity of 32‰ at 18 °C for a week before processing. *V. proteolyticus* bacteria were grown in 2216E marine broth at 28 °C. One group of clams was injected in the adductor muscle with 1 mL of live bacterial cells resuspended in sterile 0.01 M phosphate buffer solution (PBS), pH 7.5, OD_600_ = 0.4; clams from the second group were placed in an aquarium containing diesel fuel (0.5 mg/L); a third group of clams injected with *V. proteolyticus* was placed in an aquarium with diesel fuel (0.5 mg/L); and the untreated clams were used as control group. Five individuals from each group were randomly collected at 0, 0.5, 3, 6, 12, 24, and 48 h post-challenge, and hemolymph was obtained to analyze GYL expression levels by ELISA. 

### 3.16. Statistical Analysis

Experimental data are presented as mean ± SD. A *p*-value less than 0.05 was considered statistically significant. All analyses were carried out via Student’s two-tailed *t*-test.

## 4. Conclusions

Innate invertebrate immunity is a complex system that is far from fully understood. Research interest has focused on the immune response of invertebrates in the presence of bacterial infections and anthropogenic factors. Our results suggest that GYL is a key molecule involved in the modulation of this response during the simultaneous presence of both bacteria and diesel fuel, as opposed to during contamination with *V. proteolyticus* or diesel fuel separately, as these conditions did not show the same response. Furthermore, these results suggest that *G. yessoensis* may present a specific immune response depending upon the type of immunostimulant present. This property can be used for the environmental monitoring of the state of sea areas and of anthropogenic pollution of the environment.

The microbial-binding activity of GYL to Gram-positive and Gram-negative bacteria suggests interest in its use as a potential therapy for these infections. Thus, lectins are considered one of the most versatile groups of proteins used in biological processes and biomedical research.

## Figures and Tables

**Table 1:** Purification of lectin from the hemolymph of the of the mollusk *G. yessoensis*.

Sample	Volume (mL)	Titer HA	Total Activity ^a^	Protein Concentration (mg/mL)	Protein Amount (mg)	Specific Activity ^b^	Purification Ratio (fold) ^c^	Recovery of Activity (%) ^d^
Crude hemolymph	50	512	25,600	11.75	587.50	43.57	1	100
Purified lectin	2	2048	4096	0.63	1.26	3250.79	74.61	16

^a^ Total activity is calculated as titer × volume; ^b^ Specific activity is calculated as titer/mg of protein; ^c^ Purification ratio is calculated by comparing the value of specific activity of the crude extract vs. purified lectin; ^d^ Recovery of activity is calculated by comparing the value of total activity of the crude extract vs. purified lectin.

**Table 2:** Agglutination of erythrocytes by GYL.

Type of Erythrocytes	Titer of Agglutination (the Reciprocal of the of Highest Dilution of Lectin That Gave Visible Hemagglutination)	
	Untrypsinized	Trypsinized
Human O	128	256
Human A	16	16
Human B	16	16
Human AB	32	128
Rabbit	128	512
Mouse	8	32

**Table 3:** Specificity of GYL for carbohydrates and glycoproteins.

Inhibitor	Concentration at Half-Maximal Inhibition of Binding
L-Fucose	0.17 mM (0.028 mg/mL)
PSM	0.033 mg/mL
Asialo-PSM	0.008 mg/mL
Fetuin	0.008 mg/mL
Asialofetuin	0.004 mg/mL
Thyroglobulin	0.004 mg/mL
Ovalbumin	0.025 mg/mL

The following substances caused no inhibition when used at 10 mg/mL: D-glucose, N-acetyl-D-glucosamine, D-galactose (Gal), N-acetyl-D-galactosamine (GalNAc), D-mannose, N-acetyl-D-mannosamine, N-acetyl-neuraminic acid, N-acetyl-glycoloylneuraminic acid, *p*-nitrophenyl-β-D-galactoside, Galα1-3GalNAc, methyl-α-D-galactopyranoside, lactose, melibiose, ovomucoid, α1-acid glycoprotein, and mannan from *Saccharomyces cerevisiae.*

**Table 4:** Components of the secondary structure of GYL.

Sample	α-Helix			β-Sheet			β-Turn	Random Coil
	I	II	III	I	II	III		
GYL	0.1	4.6	4.7	28.3	13.7	42.0	20.7	32.6

**Table 5:** Peptide sequences.

Peptide	*m*/*z*	z	Mass
TTASQLENASKNHYWLNGTDSAVEGQFR	1042.8264	3	3125.4326
CFSYVDWMSAEEPNDRFDADCLHLR	1045.1152	3	3132.3164
WNDLSCSK	505.2257	2	1008.4335
LPFFFLCEKPTETCSDK	1060.0051	2	2117.98
MTQAAAEEYCTTQDGHLAQPTSEGLNTFLK	1110.177	3	3327.5022
